# Implication of Fructans in Health: Immunomodulatory and Antioxidant Mechanisms

**DOI:** 10.1155/2015/289267

**Published:** 2015-03-16

**Authors:** Elena Franco-Robles, Mercedes G. López

**Affiliations:** Centro de Investigación y de Estudios Avanzados del IPN, Unidad Irapuato, Km 9.6 Libramiento Norte Carretera Irapuato-León, 36821 Irapuato, GTO, Mexico

## Abstract

Previous studies have shown that fructans, a soluble dietary fiber, are beneficial to human health and offer a promising approach for the treatment of some diseases. Fructans are nonreducing carbohydrates composed of fructosyl units and terminated by a single glucose molecule. These carbohydrates may be straight or branched with varying degrees of polymerization. Additionally, fructans are resistant to hydrolysis by human digestive enzymes but can be fermented by the colonic microbiota to produce short chain fatty acids (SCFAs), metabolic by-products that possess immunomodulatory activity. The indirect role of fructans in stimulating probiotic growth is one of the mechanisms through which fructans exert their prebiotic activity and improve health or ameliorate disease. However, a more direct mechanism for fructan activity has recently been suggested; fructans may interact with immune cells in the intestinal lumen to modulate immune responses in the body. Fructans are currently being studied for their potential as “ROS scavengers” that benefit intestinal epithelial cells by improving their redox environment. In this review, we discuss recent advances in our understanding of fructans interaction with the intestinal immune system, the gut microbiota, and other components of the intestinal lumen to provide an overview of the mechanisms underlying the effects of fructans on health and disease.

## 1. Introduction

Fructans are recognized as health-promoting food ingredients. They are found in a small number of mono- and dicotyledonous families of plants, such as Liliaceae, Amaryllidaceae, Gramineae, Compositae, Nolinaceae, and Agavaceae. Various fructan-containing plant species, including asparagus, garlic, leek, onion, Jerusalem artichoke, and chicory roots, are often eaten as vegetables [1–3]. Substantial variation in chemical and structural conformations makes fructans a flexible and appealing ingredient for different dietary products such as nutraceuticals.

Inulin-type fructans (ITFs) are among the most studied; ITFs are indigestible, fully soluble, fermentable food ingredients with known prebiotic properties. ITFs are linear fructose polymers with *β*(2→1) linkages found naturally in chicory roots, wheat, onion, garlic, and other foods. In the scientific literature, ITFs are frequently referenced generically but inconsistently as “inulin,” “oligofructose” (OF), and “fructooligosaccharides” (FOS) [4].* Agave* fructans have a more complex, highly branched structure, including *β*(2→1) and *β*(2→6) linkages. Thus,* Agave* fructans can contain an external glucose, characteristic of graminans, and an internal glucose, characteristic of neofructans. For this reason, this type of fructans has been called “agavins” [5].

Fructans contribute to host health through multiple mechanisms. Fructans are selective substrates for probiotic bacteria stimulating probiotic bacterial growth, which can confer health benefits to the host through the several mechanisms, including immunomodulation [6–8]. Fructans may also act as scavengers of reactive oxygen species [9], decreasing inflammation and improving redox status. Fructans are fermented to short chain fatty acids (SCFAs), which have important implications in host health. In addition, direct interaction between fructans and intestinal immune cells has recently been suggested. The aim of this review is to summarize the latest findings on studies investigating fructans as prebiotics and to provide an overall image of the mechanisms underlying the health effects of fructans.

## 2. Fructans: Structure, Source, and Synthesis

Approximately 15% of flowering plants store fructans as reserve carbohydrates [10]. Worldwide, the most studied and marketed fructan is inulin, which is obtained primarily from chicory roots. However, some candidate fructans, such as galactooligosaccharides (GOS) derived from lactose and lactulose, have also demonstrated potential prebiotic effects [11]. In addition to chicory root, another potential fructan source includes the more recently investigated* Agave* fructans. The* Agave tequilana* Weber* azul* variety is an economically important species of* Agave* cultivated in Mexico. Because of its high inulin concentration, this variety is the only species in the Agavaceae family that is appropriate for tequila production. The high inulin concentrations, specifically in the head (pine), provide added economic and environmental value to this species of* Agave* [12].

**Figure 1:**
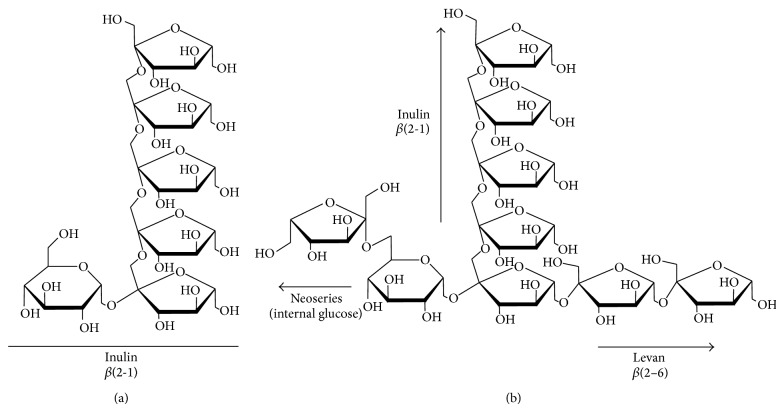
Structural comparison of the (a) inulin from* Cichorium intybus* and (b) agavin from* Agave* spp.

Fructans have been classified into 4 groups based on their structural bonds: inulin, levans, graminans, and neoseries fructans (inulin neoseries and levan neoseries mixture) [13]. Inulin is the simplest linear fructan, consisting of *β*(2→1)-linked fructose residues. Inulin is usually found in plants such as* Cichorium intybus* (15–20% fructans),* Jerusalem artichoke* (15–20% fructans),* Helianthus tuberosus* (15–20% fructans), and* Dahlia variabilis* (15–20% fructans) (Figure 1) [13–15]. Levan-type fructans (also called phleins in plants) can be found in grasses (Poaceae). Levan fructans contain a linear *β*(2→6)-linked fructose polymer and are found in big bluegrass (*Poa secunda*) [16, 17]. Graminan-type fructans consist of *β*(2→6)-linked fructose residues with *β*(2→1) branches or can consist of more complex structures in which neosugars are combined with branched fructan chains. These complex fructans are usually found in plants such as* Avena sativa*,* Lolium sp*., and* Agave sp.* (15–22% fructans) (Figure 1) [5, 18–20]. The inulin neoseries are linear (2-1)-linked *β*-d-fructosyl units linked to both C1 and C6 on the glucose moiety of the sucrose (Suc) molecule. This results in a fructan polymer with a fructose chain ((mF2-1F2-6G1-2F1-2Fn); F (fructose), G (glucose)) on both ends of the glucose molecule. These fructans are found in plants belonging to the Liliaceae family (e.g., onion and asparagus (10–15% fructans)) [15, 21]. The smallest inulin neoseries molecule is called neokestose. The levan neoseries consists of polymers with predominantly *β*(2→6)-linked fructosyl residues on either end of the glucose moiety of the sucrose molecule. These fructans are rare, although they have been found in a few plant species belonging to the Poales (e.g., oat) [18].

The length of fructosyl chains varies greatly in plants; plant fructosyl chains are much shorter than those of bacterial fructans. In general, the chain length or degree of polymerization (DP) is between 30 and 50 fructosyl residues in plants but can occasionally exceed 200 [13]. Fructans can also be classified according to their DP into small (2 to 4), medium (5 to 10), and relatively large chain lengths (11 to 60 fructose units). The term fructooligosaccharides (FOS) is used for short fructans with a DP of 3–5 derived from sucrose [22]; oligofructose (OF) is used for molecules with a DP of 3–10 derived from native inulin [23].

The biosynthesis of fructans begins with sucrose (Suc), to which fructose residues are added [4]. In plants, fructans are synthesized from Suc by the action of two or more enzymes known as fructosyltransferases. The first enzyme, 1-SST (sucrose:sucrose fructosyltransferase), initiates* de novo* fructan synthesis by catalyzing the transfer of a fructosyl residue from one Suc molecule to another, resulting in the formation of the trisaccharide 1-kestose. The second enzyme, 1-FFT (fructan:fructan 1-fructosyltransferase), transfers fructosyl residues from a fructan molecule with a DP of ≤3 to either another fructan molecule or a Suc. The actions of 1-SST and 1-FFT result in the formation of a mixture of fructan molecules with different chain lengths [13].

## 3. Functional Effects of Fructans

Worldwide, over 60% of functional food products are directed toward intestinal health, and additional therapeutic benefits of these products to human health are constantly being explored. Prebiotics are defined as “selectively fermented ingredients that allow specific changes, both in the composition and/or activity in the gastrointestinal microbiota that confers benefits upon host well-being and health” [24]. Moreover, prebiotics may suppress pathogen growth to improve overall health [25]. Current evidence indicates that beneficial bacteria reduce the risk of diseases through diverse mechanisms, including modulation of gut microbiota composition or function, and regulation of host epithelial and immunological responses. These effects may be revealed through changes in bacterial populations or metabolic activity [26]. Bacterial metabolism can confer a number of advantageous effects to the host, including the production of vitamins, modulation of the immune system, enhancement of digestion and absorption, inhibition of harmful bacterial species, and removal of carcinogens and other toxins. The resident microbiota is also known to consist of pathogens that can disrupt normal gut function and predispose the host toward disease if allowed to overgrow [27].

Fructans play protective roles in plants subjected to drought, salt, or cold stress [14]. However, the therapeutic potential of fructans in human health has only recently been explored. As described above, fructans are the most widely known and used prebiotics [28]. Of the many nondigestible food ingredients studied for their prebiotic potential, human trials favor ITFs, FOS, OF, and GOS [29–32]. Fructans have been proposed as modulators of the microbial ecology and host physiology in animals and humans [33, 34] because they are not digested [9]. Although they are subjected to minor hydrolysis in the stomach, the human gut lacks the hydrolytic enzymes capable of digesting *β* linkages [35]. Therefore, fructans reach the colon relatively intact and eventually trigger a decrease in the pH, thereby altering the colonic environment [36]. The rate and extent of ITFs fermentation appear to be strongly influenced by the DP. FOS (low DP) are rapidly fermented in the proximal colon [37], whereas inulin (high DP) appears to have a more sustained fermentation profile that potentially enables protective effects in the distal colon [4, 38]. Acting as prebiotics, inulin, FOS, and GOS improve glucose, reduce triglycerides, modify lipid metabolism, and reduce plasma LPS. Additionally, they stimulate* Lactobacillus* and* Bifidobacterium* species to reduce the presence of pathogens in the gut and relieve constipation (Table 1). Other fructans, including soluble gut oligosaccharides, mimic the sugar chains found in the glycoproteins and glycolipids of gut epithelial cells, thereby preventing the adhesion of pathogenic microorganisms [39] and exerting direct antimicrobial effects [40] (Table 1).

Interestingly, fructans from* Dasylirion *spp. (DAS) and* A. tequilana* Gto. (TEQ) increased SCFAs production and decreased colon pH in* in vitro* studies [41]. Furthermore, supplementation of the mouse diet with* Agave* fructans (TEQ and DAS) has been shown to increase secretion of GLP-1 and its precursor, proglucagon mRNA, in all colonic segments of the mouse. These results suggest that fermentable fructans of different botanical origins and with differing chemical structures are able to promote the production of satietogenic/incretin peptides in the lower part of the gut [41] (Table 1). Moreover,* Agave* fructans have been shown to have physiological effects on lipid metabolism [41, 42] and reduce oxidative stress in conjunction with phenolic compounds in* in vitro* and* in vivo* assays [42] (Table 1). For the first time, the effect of agavins from* Agave angustifolia* and* Agave potatorum* as prebiotics has been reported showing satiety effect as well as an increment on GLP-1 and a decrement on ghrelin in an animal model [43] (Table 1).

Studies have been performed to determine whether probiotics reduce cancer risk. To maximize the effect of a prebiotic compound, the prebiotic would need to ferment in the distal colon, where proteolytic fermentation predominates and toxic metabolites such as ammonia, hydrogen sulfide, and cresol are produced [44, 45]. A recent study by Gomez et al. was the first to investigate the effect of* Agave* fructan fermentation on complex fecal microbiota* in vitro* [46] (Table 1). The first clinical trial in humans with* Agave* fructans was very promising, as* Agave* treatment improved laxation [47]. Other carbohydrates, including glucooligosaccharides, isomaltooligosaccharides, lactulose, mannanoligosaccharides (MOS), nigerooligosaccharides, oat *β*-glucans, raffinose, soybean oligosaccharides, transgalactooligosaccharides, and xylooligosaccharides, are considered candidate prebiotics [31, 48]; however, more research is required.

## 4. Immunomodulatory Effects of Fructans

**Figure 2:**
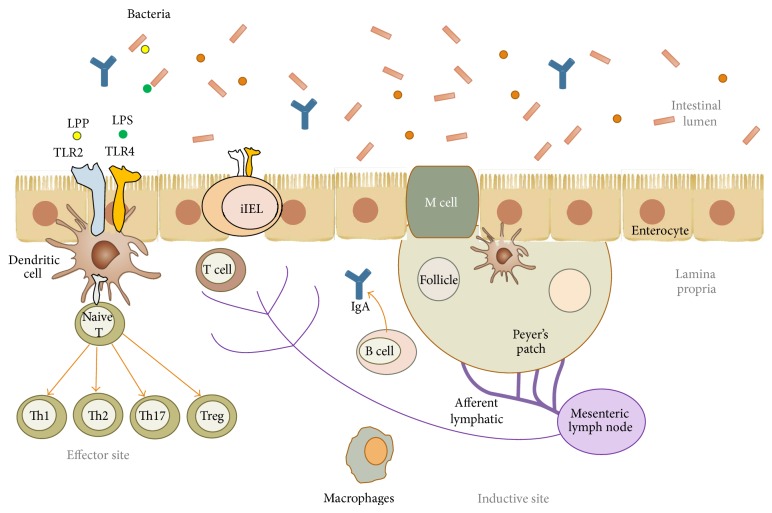
Induction of an immune response through gut-associated lymphoid tissue (GALT).

The consumption of prebiotics can modulate immune parameters in gut-associated lymphoid tissue (GALT), secondary lymphoid tissues, and peripheral circulation [70]. GALT functions to distinguish between harmful and innocuous agents and protects against infections while simultaneously avoiding the generation of hypersensitivity reactions to commensal bacteria and harmless antigens [71–73]. In inductive GALT, more structured and localized sites of antigen processing and presentation are distinguished in areas such as Peyer's patches (PPs), mesenteric lymph nodes (MLNs), the appendix, and isolated lymph nodes. GALT also contains effector sites with more diffuse organization, containing previously activated and differentiated cells that performed effector functions (Figure 2). Joint activity of the inductive and effector sites generates a rich response in immunoglobulin A (IgA) and cellular immunity, with robust cytotoxic regulatory functions and memory at the level of the mucosa and serum [74]. The intestinal epithelium provides a physical barrier that separates the trillions of commensal bacteria in the intestinal lumen from the underlying lamina propria (LP) and the deeper intestinal layers. Microfold cells (M cells), B cells (especially IgA-producing plasma cells), T cells, macrophages, and dendritic cells (DCs) in the LP are located directly below the intestinal epithelium (Figure 2). M cells are part of the epithelial layer covering the PP and specialize in transporting antigens from the lumen to GALT [75].

T and B cells are activated after initial contact with the antigen at inductive sites. These cells then proliferate, differentiate, and migrate to various effector sites, such as the LP or the intestinal epithelium, where a single population of iIELs (intestinal intraepithelial lymphocytes) and some DCs are located between the enterocytes [76–78] (Figure 2).

In fact, iIELs provide a cellular defense against any individual antigen [79]. Meanwhile, DCs are potent antigen-presenting cells critical for the induction of downstream adaptive immune responses [80]. For instance, several subsets of DCs have been identified within the PP that possess either Th1- or Th2-polarizing ability [81]. The CD103+ subset has been found within the small intestinal LP, MLN, and PP, as well as the colonic LP. CD103+ DCs have FoxP3+ Treg-polarizing ability, as well as the ability to imprint gut-homing T cells; expression of the a4b7 integrin on conventional T cells and Treg cells involved in directing gut tropism ensures their ability to be imprinted [82, 83]. CD103+ DC subsets have also been shown to induce Th17 polarization and IgA class switching [84, 85]. Moreover, all DC subsets and antigen-presenting cells, including macrophages, are equipped with a battery of pattern recognition receptors (PRRs). These receptors can detect molecular patterns of invading microorganisms or endogenous “danger” signals and stimulate the immune response. PRRs are expressed on the cell surface and intracellularly are extremely diverse and capable of detecting a wide range of molecular species, including proteins, carbohydrates, lipids, and nucleic acids [86]. The Toll-like receptor (TLRs) family is the most intensely studied family of PRRs on DCs. Triggering TLRs on DCs is thought to be critical for their functional maturation to immunogenic DCs and for their ability to prime naive T cells in response to infection. Therefore, TLR activation couples innate and adaptive immunity [87]. TLR-mediated recognition of commensal microorganisms may also play important roles in tissue homeostasis, as recent studies have shown that TLR signaling by DCs was required to maintain immune homeostasis and tolerance to gut microbiota [88]. Interestingly, Tregs are also abundant at host-microbiota interfaces. Studies have suggested that commensal microbiota can stimulate the generation of Tregs and Th17 cells [89]. These results highlight the importance of diet and the microbiota in the establishment and configuration of the immune system of the intestinal mucosa. However, whether prebiotic compounds directly affect immune components or whether they act exclusively through the modulation of the endogenous intestinal microbiota remains unclear.

### 4.1. Indirect Mechanisms of Fructan Health Effects

Prebiotics and probiotics may have indirect immunomodulatory functions through their actions on nonimmune cells, such as epithelial cells. However, they may also exert immune system-independent effects by selectively stimulating the growth and/or activity of beneficial intestinal bacteria, such as* Lactobacillus* and* Bifidobacterium* species, which results in the restoration of the normal composition of the intestinal microbiota [90]. Mutualism between the host and its microbiota is fundamental for maintaining homeostasis in a healthy individual [91]. Commensal bacteria provide the host with essential nutrients. They also metabolize indigestible compounds, defend against the colonization of opportunistic pathogens, and contribute to the development of intestinal architecture in addition to stimulating the immune system [92]. In fact, intestinal immune and metabolic homeostasis in mammals is largely maintained by interactions between the gut microbiota and GALT [93]. The host actively engages the gut microbiota and controls its composition by secreting antimicrobial peptides and immunoglobulins. Conversely, commensals shape the gut-associated immune system by controlling the prevalence of distinct T cell populations [94].* Bacteroides fragilis* protects mice from infection by* Helicobacter hepaticus* through several immunological mechanisms, including suppression of IL-17 production [95]. These commensals also express capsular zwitterionic polysaccharide A, which is a cognate antigen to effector CD4^+^ T cells [92]. Other zwitterionic polysaccharides, such as type 1 capsule of* Streptococcus pneumoniae*, can also modify inflammatory responses in animal models by stimulating IL-10-producing CD4^+^ T cells [96]. Moreover, bacterial symbionts, such as* Bacteroides*,* Barnesiella*, and* Turicibacter*, interact with CD8^+^ cytotoxic T cells in the mucosal compartment of the small intestine and colon [97].

**Figure 3:**
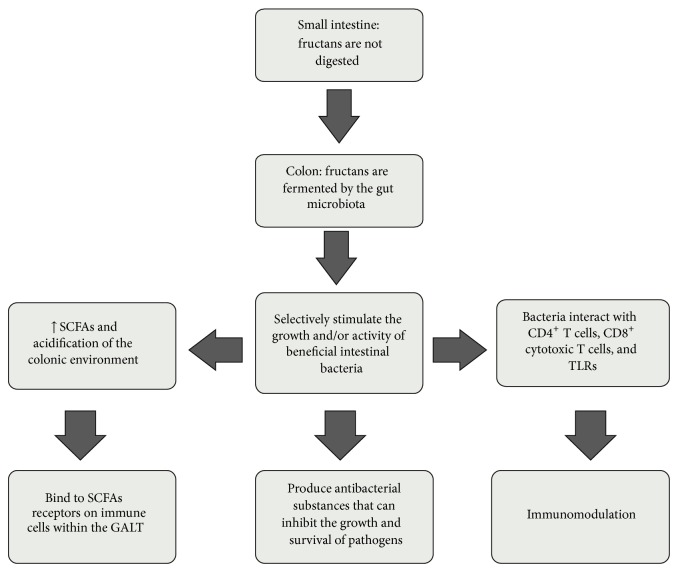
Mechanism for the indirect effect of fructans on the immune system.

Other indirect pathways by which fructans exert immunomodulatory effects include the production of SCFAs, which are the fermentation products of fructans. Inulin fermentation increases the production of SCFAs (acetate, propionate, and butyrate), lactic acid, and hydrogen (H_2_), while decreasing the pH of the colonic environment [36].* Bifidobacterium *species are able to use some monosaccharides in a unique manner to ultimately generate SCFAs [98] and acidify the colonic environment. The increase in SCFAs antagonizes the growth of some pathogenic bacterial strains [99] and favors mucin production in the colon [100]. SCFAs bind to SCFAs receptors on GALT immune cells [101–103], activating G protein-coupled receptors (GPR) [104], such as GPR41 and GPR43 [101, 102, 104]. This binding affects the recruitment of leukocytes to inflammatory sites [105, 106] and suppresses the production of proinflammatory cytokines and chemokines [106–108]. GPR43 is highly expressed in polymorphonuclear cells (PMNs, i.e., neutrophils) and is lowly expressed in peripheral blood mononuclear cells (PBMCs) and purified monocytes. Conversely, GPR41 is expressed in PBMCs but not in PMNs, monocytes, or DCs [102]. Importantly, butyrate decreases the glutamine requirement for epithelial cells and alters epithelial cell gene expression [71, 109]. The mechanism for the indirect effect of fructans on the immune system is shown in Figure 3.

### 4.2. Direct Mechanism: Pattern Recognition Receptors

In addition to the indirect effects of fructans and their fermentation products on the microbiota, the direct effects of fructans on the signaling of immune cells have gained attention as an additional pathway of immunomodulation. ITFs have been reported to interact directly with GALT components, such as gut dendritic cells (DCs) and intraepithelial lymphocytes (iIELs), through receptor ligation of PRRs [7]. Signaling through PRRs, such as TLRs (Toll-like receptors), is considered the starting point of innate immune system activation against various environmental factors, including microbes and antigens. The innate immune system enables appropriate adaptive immune responses to be generated through the activation of multiple specific immunocompetent clones [110]. TLRs play an important role in initial innate immune responses, which includes cytokine synthesis and activating acquired immunity. The *β*(2→1)-linked fructans can provide a direct signal to human immune cells primarily by activating TLR2 and to a lesser extent TLR4, TLR5, TLR7, TLR8, and NOD2. *β*(2→1)-linked fructans stimulation results in NF-*κ*B/AP-1 activation, further suggesting that *β*(2→1)-fructans are specific ligands for TLR2. However, chain length is important for the induced activation pattern and IL-10/IL-12 ratios stimulated by *β*(2→1)-fructans [111, 112]. In fact, ITFs increase the proportion of DCs in PPs and increase the secretion of IL-2, IL-10, and interferon-*γ* from the spleen and MLNs. Additionally, ITFs reduce the number and proportion of T cell receptor (TCR-) *αβ* ^+^ CD8^+^ cells in the spleen and CD45RA^+^ cells in the MLNs [113] (Table 2). Furthermore, TLR4 appears to be involved in levan *β*(2→6)-fructans pattern recognition. Oral administration of levans* in vivo* significantly reduced IgE serum levels and Th2 response in mice immunized with ovalbumin [8].

A fructose receptor may exist on immune cells, as receptors for *β*-glucan [114] and mannose [115] have been identified on the surface of immune cells. Oligofructose has also been shown to bind to receptors on pathogenic bacteria, preventing them from attaching to the epithelial membrane [116]. Furthermore, ITFs treatment of gut epithelial cells can modulate the innate immune barrier by modifying the integrity of epithelial tight junctions or by altering signals from the epithelial cells to the underlying immune cells [117].

Thirty-six fructan studies reporting immune outcomes have been conducted in mice, rats, pigs, dogs, and humans, and these investigations are summarized in Table 2. These reports show that fructans may have specific effects on different immune system components.

## 5. Fructans Act as ROS Scavengers

Because inulins and agavins have health benefits, improve blood metabolic parameters [41, 52], reduce colonic pH [152], increase SCFAs production [36, 43, 69], and stimulate the immune system [48], interest has developed in the antioxidant capacity of fructans. As in plants, fructans and other carbohydrates have been shown to scavenge ROS [153–157]. ROS include free radicals such as the superoxide anion (O_2_^•−^), hydroxyl radical (^•^OH), and nonradical molecules such as hydrogen peroxide (H_2_O_2_) and singlet oxygen (^1^O_2_). These molecules attack DNA, lipids, and proteins resulting in cellular damage [158]. Fructans, galactooligosaccharides (GOS), arabinoxylans, *β*-glucans, and fructooligosaccharides (FOS) might act as ROS scavengers in plants [159] because they have strong antioxidant activity* in vitro*. Raffinose appears to be a moderate ROS scavenger [160].

Recently reports have suggested that fructans possess antioxidant activity in* in vivo* models. A putative role for oligofructoses in counteracting the prooxidative effects of a high fructose diet has been demonstrated in rats. The addition of fructans to the diet may provide an early defense against oxidative stress and may act before the activation of the endogenous ROS detoxification systems [65]. In an indirect mechanism, these nondigestible carbohydrates might serve as ROS scavengers, which suggests that inulin can protect the colonic mucosa by acting as a barrier against oxidative stress in addition to its positive prebiotic effect. This hypothesis is consistent with the recently proposed ROS scavenging capability of inulin [65, 161] and the reported effects of SCFAs, which induce the expression of crucial antioxidant enzymes, such as glutathione S-transferases (GSTs) [162]. Li et al. showed that, in aged mice, synthetic oligosaccharides increase the activity of antioxidant enzymes [161]. By contrast, oligofructose has been shown to reduce the expression of NADPH oxidase in the colons of obese mice [51]. Moreover, intraperitoneal administration of synthetic oligosaccharides stimulates a dose-dependent decrease in lipid peroxidation, which supports the* in vivo* ROS scavenging capability of certain sugars [161]. Furthermore, agavins from* Agave tequilana* have been shown to improve the redox status in hypercholesterolemic mice by reducing malondialdehyde serum levels and oxidative protein damage. These results could be attributed to a reduction in the generation of oxidative products during digestion and colonic fermentation [42]. Additionally, polyphenol studies have indicated that metabolism in the large intestine is positively affected by prebiotic fructooligosaccharides, which have a synergistic effect with polyphenol to counteract oxidative stress in* in vivo* models [163].

## 6. Conclusion

Prebiotic consumption is undoubtedly associated with several health benefits. In this review, we assessed the potential immunomodulatory and antioxidants mechanisms of the prebiotic fructans as well as the impact of fructans on immune health. Some preliminary data have convincingly suggested that fructan consumption can modulate immune parameters in GALT. Additionally, fructans may act as ROS scavengers providing an increase in antioxidant defenses partially through the activation of endogenous ROS detoxification systems. Further studies will be required to fully understand and elucidate the mechanisms of action for fructans on GALT in various disease models.

## Figures and Tables

**Table 1:** Main prebiotic effects of fructans in *in  vitro* and *in  vivo* studies.

Effect	Type of fructan	Dose/duration	Model	Results	Reference
Decreasing blood glucose	FOS, inulin	8 g/d for 14 days; 10% for 4 weeks	Diabetic subjects; animal models	Significant reduction of mean fasting blood glucose levels. Improving glucose tolerance	[49–51]

Reduction in blood serum triacylglycerol levels	FOS, inulin	4–34 g/d for 21–60 days; 10% for 3–5 weeks	Healthy humans; obese animal models	Significant reduction in blood serum triacylglycerol levels	[52–54]

Improved lipid metabolism	FOS, GOS, inulin, and agavins	5%–10% for 21 day to 8 weeks	Obese animal models	Decrease in body weight gain. Decrease in epididymal adipose tissue, inguinal adipose tissue, and subcutaneous adipose tissue. Reducing fat-mass development	[41, 50, 51, 55–59]

Stimulation of lactobacilli and bifidobacteria and decreasing pathogens	FOS, GOS, and inulin	2.5–34 g/d for 14–64 days	Healthy subjects and animal models	Stimulating the growth of bifidobacteria and contributing to the suppression of potential pathogenic bacteria	[46, 60, 61]

Relief of constipation	Inulin, FOS, and GOS	20–40 g/d for 19 days	Constipated humans and animal models	Inulin showing a better laxative effect than lactose and reducing functional constipation with only mild discomfort	[62, 63]

Increased production of SCFAs and decreasing colon pH	Inulin, FOS, and agavins	24 g/d for 5 weeks; 10% for 28 days	Healthy subjects; animal models	Significant increase of acetate, propionate, and butyrate. Significantly increasing activity of bacterial enzymes and decreasing the pH of digesta	[36, 64, 65]

Improving mineral uptake	Inulin, FOS, and agavins	1–40 g/d for 9 days; 50–100 g/kg diet for 4 weeks	Male healthy adolescents; animal models	FOS stimulating fractional calcium absorption in male adolescents. A combination of different carbohydrates showing synergistic effects on intestinal Ca absorption and balance in rats	[66–69]

Regulated gut peptides	Inulin, FOS, and agavins	24 g/d for 5 weeks; 10% for 5 weeks	Healthy subjects; animals models	Increasing plasma glucagon-like peptide-1 (GLP-1) concentrations and reducing ghrelin. Increasing endogenous GLP-2 production and consequently improving gut barrier functions	[36, 41, 50, 57, 59]

Reducing body weight and energy intake	Agavins	10% for 5 weeks	Male healthy animal model	*Agave* fructans showing indications of prebiotic activity, particularly in relation to satiety and GLP-1 and ghrelin secretion. In this same study, the levels of butyric acid were higher for *Agave potatorum* fructans	[43]

Growth inhibition and prevention of adhesion of pathogenic microorganisms	FOS	170 mg/kg, 2 weeks of lactation	Breast-fed infant; cocultures of *Pseudomonas aeruginosa *	Oligosaccharides in human milk interfering with microbial adhesion. Reduction of exotoxin A in cultures of *P. aeruginosa *	[39, 40]

Reduction of oxidative stress by reducing ROS levels	FOS, agavins	10% for 4–8 weeks	Male obese animal models	FOS reducing TBARS urine. Lipopolysaccharides reduction in plasma. Improving the redox status by reducing the malondialdehyde serum levels and protein oxidative damage	[9, 42, 65]

Stimulation of the immune system	FOS, GOS, and inulin				See Table 2

FOS: fructooligosaccharides; GOS: galactooligosaccharides; SCFAs: short chain fatty acids.

**Table 2:** Effect of fructans on the immune function in healthy animal and human models.

Effects of fructans	Dose fructan/duration	Model	Reference
↑ DC and γδ T cells in lamina propria of the caecum and ↓ PGE2 in small intestine, colon, and caecum	3% FOS for 12 days	Mice treated with antibiotics and conventionalized with *Clostridium difficile *	[118]

In peripheral blood: ↑ CD4^+^/CD8^+^ ratio and ↓ B cells. In GALT: ↑ proportion of CD4^+^ cells and CD8^+^ cells, PP, and lamina propria cells and ↓ CD4^+^/CD8^+^ ratio in lamina propria	0.87% FOS for 14 days	Adult dogs	[119]

Synbiotics ↑ whole blood phagocyte activation level.	1% FOS for 28 days	Piglets infected with *S. typhimurium *	[120]

↑ counts of leucocytes, lymphocytes, neutrophils, CD2^+^ T cells, CD4^+^ T cells, CD8^+^ T cells, B cells, and macrophages in blood, ↑ % phagocytic activity of leucocytes and neutrophils in blood.	3 g/d OF for 20 days	Newborn piglets	[121]

↑ ileal IgA concentration.	2 g/d FOS and/or MOS for 14 days	Adult dogs	[122]

↓ blood neutrophils and ↑ blood lymphocytes.	2 g FOS plus/1 g MOS for 14 days	Adult dogs	[123]

↑ rotavirus-specific IgA levels in serum and ↓ duration of a strong rotavirus-specific IgA response in faeces and % IgA and IgG positive B cell in the PP. ↑ serum rotavirus-specific IgG and Rhesus rotavirus antigen concentration in stools.	1.25 g/L OF for 7 weeks	Mice (pups) infected with *Rhesus* rotavirus	[124]

No change in protein, alb, serum Ig, secreting IgA, and IL-4 and IFN-*γ* secretion, ↑ antibodies against influenza B and pneumococcus.	6 g OF/ITFs for 28 weeks	Healthy elderly (>70 years)	[125]

↑ % CD4 and CD8 lymphocytes, ↓ phagocytic activity in granulocytes and monocytes and IL-6 mRNA expression in PBMCs.	8 g/day FOS, 3 weeks	Nursing home elderly (77–97 years)	[126]

↑ total faecal IgA, size of PP, total IgA secretion by PP cells and IL-10 and IFN-*δ* production from PP CD4^+^ T cells.	0–7.5% FOS for 6 weeks	Female mice	[127]

↓ leucocyte counts, ↑ NK activity of splenocytes and peritoneal macrophage phagocytosis of *Listeria monocytogenes*.	2.5–10% FOS or OF for 6 weeks	Female mice	[128]

↑ total number of immune cells in PP, B lymphocytes in PP and T lymphocytes and CD4^+^/CD8^+^ ratio in PP in endotoxemic mice only.	10% FOS for 16 days	Female mice healthy or endotoxemic	[129]

↓ peripheral blood lymphocyte concentration.	1% ITFs/MOS for 4 weeks	Senior dogs	[130]

↑ total intestinal IgA, ileal and colonic polymeric Ig receptor expression, ileal IgA secretion rate, IgA response of PP cells, and % of B220^+^ IgA^+^ cells.	5% FOS for 23–44 days	Newborn mice	[131]

↑ IL-10 and IFN-*δ* production in PP, secretory IgA concentration in ileum and caecum.	10% FOS-enriched ITFs for 4 weeks	Male rats	[132]

↑ NK activity. Prevention of the decrease in proportion of T cells with NK activity.	6 g/d OF and ITFs (2 : 1 ratio) for 1 year	Elderly free-living adults (age ≤ 70 years)	[133]

Improved response to some vaccine components and increased lymphocyte proliferation to influenza vaccine components.	4.95% FOS for 183 days	Healthy adults (age ≤ 65 years)	[134]

↑ T cells, MHCII on antigen-presenting cells in spleen, MLN, and thymus, IL-2 and IL-4 in blood.	10% FOS/ITFs for 4 months	Male rats	[135]

Trend towards higher fecal sIgA.	0.6 g (GOS/FOS)/100 mL formula for 32 weeks	Newborn non-breast-fed infants	[136]

Improved response to ↑ B cells, ↓ memory cytotoxic T cells, ↑ influenza-activated lymphocytes (CD69 and CD25) and IL-6 and ↓ IL10.	4.95% FOS for 4 weeks	Healthy adults (age ≤ 65 years)	[137]

In pregnant females and pups no effect on serum IgG1, IgG2, IgA, or IgM. In colostrum and milk ↑ IgM.	0.1% OF during lactation	Pregnant female dogs and pups	[138]

↓ severity of enterocyte sloughing.	1% FOS or ITFs for 14 days	Puppies	[139]

↑ % CD19 (B) cells, CD3^+^ HLA-DR^+^ (activated T cells) and ↓ % ICAM^−1^ bearing lymphocytes and % CD3^+^ NK^+^ cells.	9 g/d ITFs for 5 weeks	Adults smokers and nonsmokers	[140]

↑ vaccine-specific faecal IgA and plasma IgG levels, peritoneal macrophage activity, mean fluorescence intensity of MHCII^+^ cells in spleen, IL-12 and IFN-*δ* production by splenocytes, and survival from *Salmonella* infection when given vaccine.	5% mix (ITFs, FOS, and OF) for 1 week	Female mice	[141]

↑ fecal sIgA.	6 g/L GOS/FOS (9 : 1) for 26 weeks	Newborn healthy infants	[142]

↑ NK activity, and IL-10, ↓ IL-6, IL-1β, and TNF-α.	5.5 g GOS/d for 10 weeks	Elderly (64–79 years)	[143]

↑ DCs in PP, ↑ IL-2, IL-10, and IFN-δ from spleen and MNL cells. ↓ number and proportion of T cell receptor (TCR-) αβ^+^CD8^+^ cells in spleen and CD45RA^+^ cells in MLN.	5% ITFs for 4 weeks	Female rats	[113]

↓ total IgE, IgG1, IgG2, and IgG3; ↓ cow's milk protein-specific IgG1.	8 g/L GOS/FOS for 6 months	Newborn infants at risk for allergy	[144]

↓ intestinal sIgA.	2.51–0.42 g/kg/d mix of GOS, XOS, OF, and ITFs (3.6 : 1 : 0.4 : 5) for 12 days	Female rats induced with diphenoxylate	[145]

↓ IL-1*β* in macrophage cultures and ↑ fecal IgA.	3–5% FOS for 30 days	Female mice	[146]

↓ LPS in blood and ↓ LPS-induced increases in gene expression in IL-1*β* and LPS-induced decreases in gene expression in IL-13 in blood.	5 g XOS, ITFs–XOS (3 : 1) for 4 weeks	Healthy volunteers	[147]

↓ serum cortisol, TNF-*α* and IL-6 after a LPS injection.	0.10% levan-type fructan for 42 days	Growing pigs	[63]

↑ fecal secretory IgA and ↓ fecal calprotectin and plasma C-reactive protein.	5.5 g/d B-GOS (Bi^2^muno) for 12 weeks	Overweight adults	[148]

↑ TGF-*β* secretion by splenocytes and IFN-*γ* production and ↓ IL-5.	GOS/ITFs (dose and duration data not shown)	Healthy mice	[149]

↓ CD16/56 on natural killer T cells and ↓ IL-10 secretion, XOS and Bi-07 supplementation ↓ CD19 on B cells.	8 g XOS or with 10^9^ CFU Bi-07/d for 21 days	Healthy adults (25–65 years)	[150]

↑ cell-mediated immunity in terms of skin indurations and CD4^+^ T-lymphocyte population.	20–60 g/kg FOS/ITFs for 12 weeks	Healthy rats	[151]

FOS: fructooligosaccharides; PGE2: prostaglandin E2; GALT: gut-associated lymphocyte tissue; CD: cluster of differentiation; PP: Peyer's patch; OF: oligofructose; MOS: mannanoligosaccharides; IgA: immunoglobulin A; IgG; immunoglobulin G; ITFs: inulin-type fructan; IL: interleukin; PMBCs: peripheral blood mononuclear cells; NK: natural killer cells; MHC II: major histocompatibility complex II; GOS: galactooligosaccharides; HLA: human leukocyte antigen; ICAM-1: intercellular adhesion molecule 1; IFN-*γ*: interferon gamma; DC: dendritic cell; TCR: T cell receptor; MLN: mesenteric lymph nodes; XO: xylooligosaccharides; LPS: lipopolysaccharides.
